# Application of Laser-Induced, Deep UV Raman Spectroscopy and Artificial Intelligence in Real-Time Environmental Monitoring—Solutions and First Results

**DOI:** 10.3390/s21113911

**Published:** 2021-06-05

**Authors:** Claudia Post, Simon Brülisauer, Kryss Waldschläger, William Hug, Luis Grüneis, Niklas Heyden, Sebastian Schmor, Aaron Förderer, Ray Reid, Michael Reid, Rohit Bhartia, Quoc Nguyen, Holger Schüttrumpf, Florian Amann

**Affiliations:** 1 Department of Engineering Geology and Hydrogeology, RWTH Aachen University, Lochnerstr. 4-20, 52064 Aachen, Germany; luis.grueneis@rwth-aachen.de (L.G.); niklas.heyden@rwth-aachen.de (N.H.); sebastian.schmor@rwth-aachen.de (S.S.); aaron.foerderer@rwth-aachen.de (A.F.); amann@lih.rwth-aachen.de (F.A.); 2 Artha, Wagistrasse 21, CH-8952 Schlieren, Switzerland; 3 Institute of Hydraulic Engineering and Water Resources Management, RWTH Aachen University, Mies-van-der-Rohe-Str. 17, 52056 Aachen, Germany; waldschlaeger@iww.rwth-aachen.de (K.W.); sekretariat@iww.rwth-aachen.de (H.S.); 4 Photon Systems Inc., 1512 Industrial Park St., Covina, CA 91722-3417, USA; w.hug@photonsystems.com (W.H.); r.reid@photonsystems.com (R.R.); m.reid@photonsystems.com (M.R.); r.bhartia@Photonsystems.com (R.B.); q.nguyen@Photonsystems.com (Q.N.)

**Keywords:** environmental monitoring, machine learning, data processing, real-time water analysis, DUV Raman/fluorescence spectroscopy

## Abstract

Environmental monitoring of aquatic systems is the key requirement for sustainable environmental protection and future drinking water supply. The quality of water resources depends on the effectiveness of water treatment plants to reduce chemical pollutants, such as nitrates, pharmaceuticals, or microplastics. Changes in water quality can vary rapidly and must be monitored in real-time, enabling immediate action. In this study, we test the feasibility of a deep UV Raman spectrometer for the detection of nitrate/nitrite, selected pharmaceuticals and the most widespread microplastic polymers. Software utilizing artificial intelligence, such as a convolutional neural network, is trained for recognizing typical spectral patterns of individual pollutants, once processed by mathematical filters and machine learning algorithms. The results of an initial experimental study show that nitrates and nitrites can be detected and quantified. The detection of nitrates poses some challenges due to the noise-to-signal ratio and background and related noise due to water or other materials. Selected pharmaceutical substances could be detected via Raman spectroscopy, but not at concentrations in the µg/l or ng/l range. Microplastic particles are non-soluble substances and can be detected and identified, but the measurements suffer from the heterogeneous distribution of the microparticles in flow experiments.

## 1. Introduction

Repeated sampling of the aquatic environment is a fundamental component of environmental research [1]. Especially for pollutants whose concentrations should remain low or are to be reduced in the future, monitoring is indispensable, as this is the only way to detect changes in environmental pollution over time and to quantify the effect of measures and management strategies [1,2]. Examples of such monitoring activities are (1) the repeated quantification of nitrate and nitrogen levels in the fluvial environment [3], (2) the frequent quantification of pharmaceuticals, bacteria or viruses in the fluvial environment or in the effluent of wastewater treatment plants [4,5,6], and (3) the systematic quantification of plastic waste in the marine and fluvial environment [7,8,9].

The European Water Framework Directive (WFD) and the Marine Strategy Framework Directive (MSFD) request a monitoring program to examine the quality of rivers and oceans. The WFD demands regular measurements of a variety of substances, currently excluding pharmaceuticals and microplastics. However, the need for sustainable environmental management and a growing awareness of the potential negative impact of micropollutants on ecosystems requires including these substances in the WFD and the MSFD [10]. Based on the requirements of the WFD/MSFD and the emerging need for an extended list of micropollutants for future monitoring activities, a variety of analytical methods exist [11,12,13]. However, the conventional methods are time-consuming (e.g., due to sample preparation requirements), selective, susceptible to ionic interference, or require expensive sorbents that may be harmful to the environment [11]. It is therefore desirable to have a measuring device that can measure and identify all substances at the same time, without using environmentally harmful and expensive chemicals.

Raman spectroscopy has been widely deployed over the past years. For example, surface-enhanced Raman spectroscopy (SERS) seems to be a promising method for water monitoring [14,15,16,17,18,19,20,21,22]. However, limitations still exist with respect to the detectability of Raman-inactive substances as well as the superposition of Raman signals by fluorescence, suggesting a combination of both detection methods to become more flexible in application.

Thus, in the future, a methodology will be required that enables detection and quantification of an increasing variety of pollutants in aquatic systems, often in very harsh environmental conditions as is typically found in wastewater treatment plants, oceans, lakes and other fluviatile systems. In our experiments, we show the detection ability of the Raman spectrometer for nitrates, some pharmaceuticals, and microplastics and their additives.

The newly developed deep-UV Raman/fluorescence spectroscope presented in this paper shows the potential to monitor a variety of substances without the need for sample preparation. The collected Raman spectra are analyzed with the use of a pre-trained convolutional neural network (CNN), which has proven to be a reliable tool for the classification of spectral data [23]. This offers the ability for the automatic detection of chemicals in real-time, enabling reliable online measurements. In the following, the technical principles and first applications of the sensor system are described. The contribution of the paper offers the potential of Raman spectroscopy to detect substances down to a specific detection limit with several limitation factors, photo-degradation effects, spectral masking of solvents, and flow velocity in a flow cell. In this study, detection limits were estimated for nitrate/nitrite, selected pharmaceuticals, and the most widespread microplastic polymers as accuracy, precision, and device-specific sensitivity were quantified. In a companion paper, we extend the analysis to laser-induced fluorescence spectroscopy (LIF).

## 2. Laser-Induced DUV Raman/Fluorescence Spectroscopy

The environmental monitoring of chemical substances in aquatic systems is challenging since the water is a complex mixture of dissolved chemicals and suspended solids. In our current research, we investigate both Raman and fluorescence spectroscopy conducted with excitation in the deep UV below 250 nm since both methods are complementary and orthogonal and provide different but important information about materials within water. Deep UV excited Raman provides information about the molecular resonances of materials within the laser excitation volume, while fluorescence provides information about the overall electronic structure of the materials in the same laser excitation volume, as long as the excitation wavelength is sufficiently below the fluorescence features of interest to separate these two detection modes. Fluorescence background obscuration or interference of Raman emissions is a serious limitation for a wide range of analytes and background materials [24] when excitation occurs at wavelengths above 250 nm, whether in the near UV, visible, or IR [20,25,26]. A unique property of fluorescence is that over 95% of all organic materials do not fluoresce below a wavelength of about 270 nm, independent of excitation wavelength. As a result, if excitation occurs below about 250 nm, there is a fluorescence-free spectral region in which weak Raman emissions can be detected [25]. Since the spectral regions of Raman and fluorescence do not overlap, a Raman-free spectral region exists for fluorescence detection also. This was first demonstrated by Asher [25] in general and by Ianoul et al. [27] in wastewaters. This spectral separation enables the Raman detection of species such as nitrates and nitrites, and other substances without the interference from organic materials found in all wastewaters. This spectral separation is important since fluorescence emissions are typically 100 thousand to 10 million times more efficient than Raman emissions so that a minute amount of fluorescent material within the view volume of the detector will obscure or alter the much weaker Raman emissions. Fluorescence detection on its own enables detection at much lower concentrations and provides the ability to also classify organic material by both phenyl-ring number and side chains, including microbial composite materials such as microbes or microplastics. Some materials do not fluoresce regardless of the excitation wavelength and are therefore only detectable with the Raman method. There are other advantages to deep UV excitation, including signal enhancement due to resonance and pre-resonance effects, which improves detection limits.

## 3. Material and Methods

**Figure 1:**
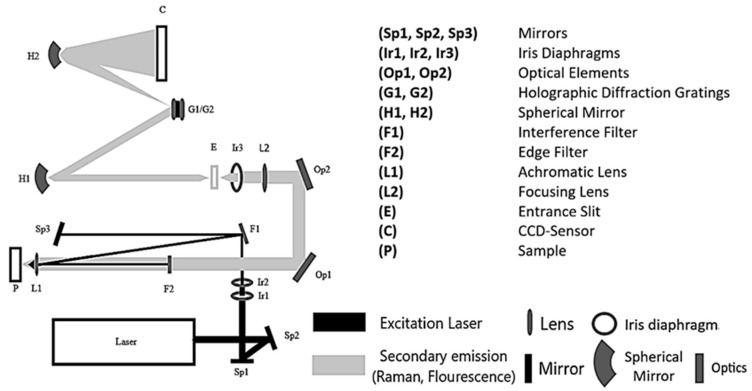
Optical set-up of the DUV Raman PL 200 from Photon Systems.

Although the benefits of deep UV Raman spectroscopy have been known for many years [25], the lasers employed up until the present are very large, expensive, need large amounts of power, and water cooling. To overcome these physical and cost limitations, we utilize a compact, low-power, air-cooled NeCu transverse excited hollow cathode gas laser with an excitation wavelength of λ = 248.6 nm in the deep UV range from Photon Systems Inc. With this excitation wavelength, the Raman signals can be observed without fluorescence obscuration or interference of Raman spectrum [28,29,30]. An independent combination of both types of emission can improve the structural elucidation of unknown substances and show their substance-specific spectra without interference or obscuration [30,31]. All Raman measurements were performed using a commercially available Photon Systems’ Deep UV Raman and Photoluminescence 200 (RPL 200; https://photonsystems.com/products/lab-spectrometer-systems/raman-pl200/, accessed on 28 May 2021) instrument (Figure 1). The RPL 200 employs a 248.6 nm NeCu laser, a 200 mm focal length Czerny–Turner spectrometer with computer-controlled 3600 and 300 g/mm holographic grating, and a 2048 × 128 pixel, three-stage thermo-electrically cooled, back-thinned, back-illuminated CCD array detector. This fully self-contained portable instrument weighs about 12 kg. The detector provides highly sensitive data (16 Bit CCD sensor resolution) with a very good spectral resolution of close to 25 1/cm to enable the detection of even small changes in the composition and or concentration of the dissolved substances. The resolution and LOD (limit of detection) are coupled via the slit size used: a larger slit leads to a better LOD but a lower resolution and vice versa. Since we were mostly interested in an improved LOD, we used a larger slit of 150 µm, which leads to a resolution of 25 1/cm. The resolution can be increased to 8 1/cm by using a 50 µm slit or even smaller to further enhance the resolution. The laser is fired in pulses with 40 µs duration and average laser energy of 3.5 µJ.

The monochromatic light of the NeCu laser is focused by two mirrors (Sp1, Sp2) to get through two iris diaphragms (Ib1, Ib2), which regulate the aperture and the light intensity. The beam passes through an interference filter (F1), coated with a dielectric material, allowing the reflection of only the laser excitation wavelength of λ = 248.6 nm. After that, the light is directed to a third mirror (Sp3) and then thrown back onto the interference filter (F1). The laser beam gets reflected at a different angle onto the achromatic objective (L1), which focuses the beam onto the measuring cuvette or the sample (P). The scattered Raman radiation passes through the achromatic objective (L1) and a lens (F2), which correspond to an edge filter. The Raman radiation is guided through the entrance slit (E) via two optical elements (Op1, Op2), a focusing lens (L2), and an iris diaphragm (Ib3) to finally hit the two spherical mirrors (H1, H2). The Raman radiation gets scattered onto one of the two holographic diffraction gratings (G1, G2) to be detected by the CCD sensor (C).

To prove the usability of the deep UV laser-induced Raman for environmental monitoring, we executed a number of laboratory tests to investigate influencing factors, such as the traceability of the substances, optical settings, agglomeration of insoluble substances, and flow velocity in a flow test. We focus on three groups of substances based on their environmental impact and their current scientific significance, namely nitrate/nitrite, selected pharmaceuticals, and microplastics.

The first step of the experimental methodology is the measurement of the pure substances (with 10 times repetition for accuracy studies) in a fused silica cuvette. This aims to specify the pure substance signal with the highest possible intensity, with an optimal sample distance to get a reference signal for recognition. In the second step, a dilution series from the substances was performed to reach the detection limit in the cuvette and to separate the signal of the fused silica cuvette from the substance signal. Consequently, in the case of substance solubility in water, the water spectrum has to be subtracted from the received signal, whereas the same procedure has to be performed when substances are only soluble in ethanol. In this setting, the influence of solvents was investigated regarding different solution concentrations on the course of the spectrum. In the third step, substance recognition in a flow cell with a sapphire lens was conducted, limited to water-soluble substances or microplastics. The objective of this set-up was to study the optical interferences of different glass types and to evaluate flow velocities and concentrations at which the detection of plastic particles was still possible.

### 3.1. Substances of Interest

Nitrate/Nitrite, Pharmaceutical Micropollutions, Microplastic (MP)

Nitrate (NO_3_) has strict threshold values (<50 mg/l), which must be maintained in most European countries’ groundwaters (Nitrates Directive (91/676/EEC), Groundwater Directive, Water Framework Directive). Heavily loaded groundwater under farmland may reach up to 4000 mg/l of nitrate [32] due to over-fertilization. The EU groundwater guideline from 2006 (118/EG) demands monitoring the chemical status for assessing groundwater resources and quality. For detection of nitrate and nitrite ions, we used IC standard solutions by companies Merck and CarlRoth, with a concentration of 1000 mg/l. The nitrate standard solution is certified and traceable to NIST (National Institute of Standard and Technology) standard reference materials, and it is manufactured in an ISO 17,034 accredited environment. The Nitrite Standard Solution is traceable to SRM from NIST NaNO_2_ in H_2_O 1000 mg/l NO_2_ Certipur^®^ (company Merck).

**Figure 2:**
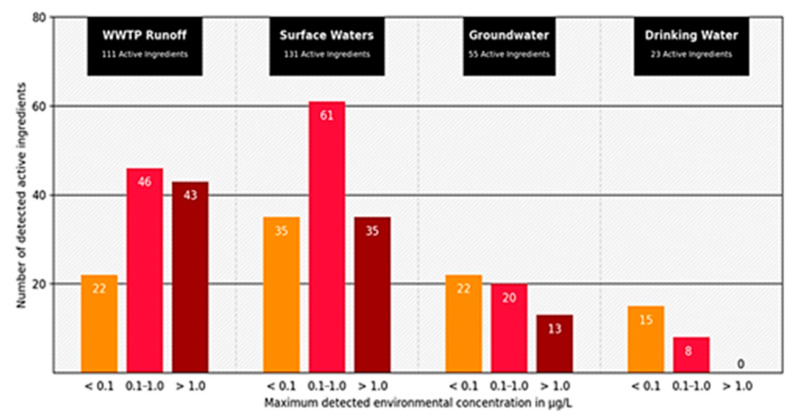
Number of active pharmaceutical ingredients measured in wastewater treatment plant effluents (111 active ingredients), surface (131 active ingredients), ground (55 active ingredients), and drinking water (23 active ingredients). Displayed according to concentration classes (<0.1 µg; 0.1–1.0 µg; >1.0 µg) of the maximum measured concentration (modified according to [35] download available at https://www.umweltbundesamt.de/publikationen/zusammenstellung-von-monitoringdaten-zu (accessed on 3 June 2021).

The concentrations of human pharmaceuticals in wastewater are usually well below critical thresholds, but adverse effects of pharmaceuticals were detected and proven [33]. The influence of antibiotics in wastewater on the development of bacterial resistance is also a crucial point. This topic is becoming increasingly important due to the partial degradability of the drugs in sewage treatment plants (Figure 2), incomplete absorption in the organism, and increasing prescription quantities [34].

To date, the most common techniques for the determination of drugs in wastewater for sewage treatment plants are liquid chromatography or gas chromatography in combination with mass spectrometry, as they can detect substances down to the ng/L range [36].

In this study, acetaminophen (also sold under the brand paracetamol, purity > 99%), metformin hydrochloride (treatment of diabetes, purity > 99%), carbamazepine (anticonvulsants, treatment of epilepsy, purity > 98%), hydrochlorothiazide (diuretic medication against high blood pressure, purity > 99%), naproxen (analgesic, purity > 98%), were tested. All pharmaceuticals were delivered by the company BioTrend (Cologne Germany), and the purity information is according to the manufacturer.

For this study, we utilize microplastic species, which are typically found in the aquatic environment, polyethylene (PE), polypropylene (PP), polyvinyl chloride (PVC), polystyrol (PS), PC (polycarbonate), PES (polyester), PET (polyethylene terephthalate), PUR (polyurethane), PA (polyamide) and HDPE (high-density polyethylene) [37]. The recyclates were provided by the manufacturer Tetralog Kunststoffrecycling e.K, which produces plastic regrind, pellets, and granules on six lines, including two dedusting systems and one screening plant [38].

### 3.2. Measurement, Preparation, and Experimental Strategy

For the measurement of the above-mentioned substances, we used commercial high purity substances in order to avoid unintended additives or contaminations. The accuracy of the spectrometer is determined by acetonitrile (ACN). ACN is also used as the calibration standard, which is listed by the ASTM (American Society for Testing and Materials) as a calibration standard [24]. The wavenumbers (1/cm) of three Raman peaks, 1376 1/cm, 2949 1/cm, 2999 1/cm, from the NIST (National Institute for Standards and Technology), are used as a reference for calibration (NIST). The precision of the Raman bands was assessed based on repeated (i.e., 10) measurements under identical settings (number of pulses; sample focal distance) and the resulting standard deviations s (Equation (1)).

(1) $$s=\sqrt{\frac{\sum_{i=1}^{n}{\left(x_{i}-\bar{x}\right)}^{2}}{n-1}}$$

With *n* being the number of measurements and $\bar{x}$ the mean value of the measurements.

All measurements were performed at room temperature.

In the first step, all pharmaceuticals were placed into the cuvette holder as an unprocessed powder. In the second step, they were brought into the solution, which was prepared with a scale and a pipette. To speed up the dissolution process, they were shaken and/or carefully warmed. Ethanol and water were used as solvents, depending on the drugs’ dissolution properties.

Nitrate and nitrite were measured in aqueous solutions with the inevitable consequence that the spectrum of the water was always recorded. Correction of the unwanted water and cuvette signal was achieved by varying the distance from the laser lens to the cuvette (sample distance) to minimize its influence since a simple “subtract” would erase the whole signal. This correction was applied to all results.

The separation of the different kinds of plastic happens to be experience-based without any technical set-up. There was no sample preparation besides washing under running water, air drying, and sieving with a sieving machine (Retsch^TM^, with sieve inserts with a mesh size of 0.063, 0.125, 0.25, 0.5, 1.0, 2.0, 4.0, 8.0 mm) to fractionate the particle size.

For the Raman static sample measurements, water samples were contained in fused silica cuvettes from the manufacturer Hellma^TM^ or Starna Cell^TM^. A cuvette containing the sample was located in front of the objective lens at the input/output of the RPL 200 instrument. Due to the fused silica of the cuvette also producing Raman emissions that can interfere with the water measurements, the 248.6 nm laser beam was focused nominally in the middle of the cuvette and not near the water/fused silica wall interface to maximize the signal from the water sample and minimize interference from the fused silica. The position of the laser focal point within the cuvette is important in minimizing this interference. Even with this minimization from the water spectral signal, the fused silica spectral background must be subtracted. For Raman flow measurements, the flow cell operates with a sapphire glass, which causes less optical interference than quartz glass.

The tests showed a signal optimum at a sample distance of 20.8 mm between the cuvette and the lens for the nitrate/nitrite measurements.

For thorough cleaning of the cuvette, the cuvette was placed in a bath of Hellmanex™ solution at approximately 40 °C for 30 min. For most experiments with water-soluble substances, thoroughly rinsing the cuvette with distilled water was sufficient.

**Figure 3:**
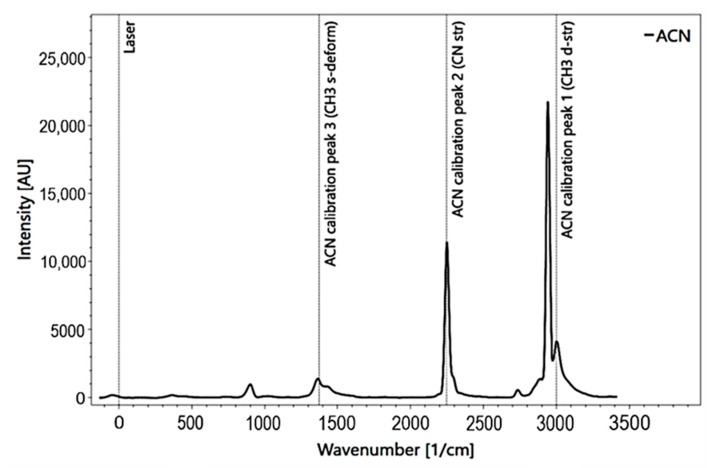
Acetonitrile calibration spectrum for Raman spectroscopy with added peak information.

The measuring process was carried out with the software Spectrum Analyzer [39]. This software allows the operating of the spectrometer and the processing of the data. The measurement set-up requires cooling the camera down to −35 °C by an internal cooling system to reduce the natural noise caused by heat. Before measurements, the RPL 200 spectrometer instrument was calibrated. Acetonitrile (C_2_H_3_N) was used for calibration at a sample distance of f = 10 [mm] and a pulse count of P = 20 (Figure 3). Based on the position of the peaks and their distance to each other, the grid was optimally positioned.

**Figure 4:**
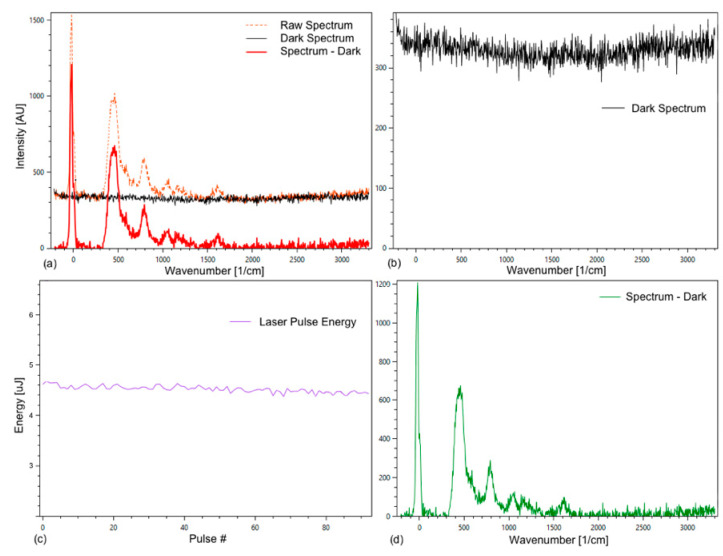
Tab Spectrum Debugger of the Spectrum Analyzer with raw, dark, and substance spectrum of an empty fused silica cuvette (**a**); (**b**) shows the dark spectrum; (**c**) energy plotted against the pulse count; (**d**) substance spectrum. The observed Raman spectrum is from the fused silica of the cuvette, which presumably is out of focus for the spectrometer, but strong enough to be visible.

To perform a Raman measurement, a grid with 3600 grid lines per millimeter was selected. For the analysis, the dark spectrum was subtracted from the raw spectrum (Figure 4).

In addition, the laser energy was recorded (Figure 4c), which enables the comparison of peak intensities of different spectra and an improved averaging of spectra. The measured laser energy was used to normalize all spectra to the same laser energy to compensate for a small deviation in the laser pulse energies. The average deviation is normally within a few percent.

The experimental procedure for nitrate/nitrite starts with the measurement of solid matter and then with solutions with concentrations of 0.5, 5, 10, and 50 mg/l. In the second step, different concentrations were measured in a flow cell using a sapphire glass lens instead of the fused silica cuvette to examine the influence of the different glass types. The pharmaceuticals, which were diluted in water or ethanol, were also first measured as a solid, pure powder. Repeated measurements were performed to identify possible photo-degradation effects. Subsequently, they were measured in solution at different concentrations (e.g., metformin hydrochloride 0.01, 0.1, 1, 10 g/l) in a fused silica cuvette (for the evaluation of the detection limit, accuracy and influence of solvent). Finally, the experiments were performed in a flow cell set-up to consider the detection limit at a distinct flow velocity. Only the microplastic recyclates were not soluble. As a consequence, concentration calculations differ from the other substances. For the detection of the MP particles within the flow cell, the measurement time is not an independent variable. However, relations between flow velocity, particle size, and concentration exist [40]. Thus, the particle concentration was quantified using an experimental approximation for determining the number of particles *N_particles_* using *ρ_material_* the density of the polymer, per particle size class from the weighed mass of the sieve residues by the respective mesh size (Equation (2)):

(2) $$N_{particle}=\frac{mass_{residues}}{V_{spherical}}\cdot \rho_{material}$$

The calculation of the volume *V_spherical_* is based on the assumption of an ideal, spherical particle. For the particle diameter, the averaged value of the two utilized sieve mesh sizes was used. With the calculated number of particles *N_particles_*, a concentration (particles per volume) can be given. Furthermore, the mass concentration was calculated [40]. The concentration of particles/m^3^ has become generally accepted [41,42], although it can also be given in g/cm^3^ according to SI conformity. In this study, we utilized both units to ensure comparability with other studies.

For the MP detection, a rotary table was used to bypass the optical interference of the fused silica cuvette. Additionally, the height of the mapping table is adjustable, so the sample can be focused on the ideal substance-specific focal length of the optics. Furthermore, the measurement within the darkened mapping device leads to a better SNR ratio. By rotating the table, the influence of the particle surface and the photodegradation could be investigated. The MP particles were positioned in the focus of the laser by using a focus plate.

To test the identification of MP in real-time, different experiments using two sizes of flow cells were carried out. For the flow cell experiment, particles larger than 0.125 mm were measured to assess the influence of measuring time, flow velocity, particle size, concentration, and their relationships. For measurements in the small flow cell, a particle size of ≥2 mm and a mass concentration of 3.3 × 10^−6^ g/ml were used. The rationale for using a large particle size was to assess the impact of stationary and unsteady flow. The number of pulses was increased gradually.

**Figure 5:**
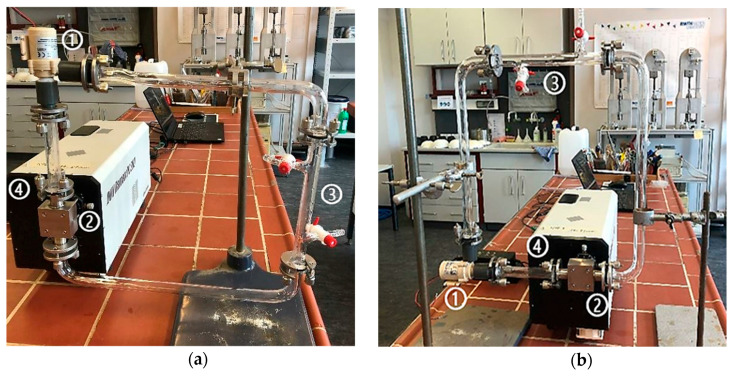
Flow cell construction in the vertical direction (**a**) and horizontal direction (**b**) with pumping, (1) pump, (2) flow cell, (3) inlet and outlet, (4) laser-spectrometer.

The first Raman measurements in the flow cells were initially carried out without the use of the pump. The MP particles should flow through the experimental set-up only due to the hydraulic potential from the much higher reservoir. However, the implementation failed due to the regulation of the flow and the surface tension, which caused the MP particles to accumulate on the glass surface. After this, the measurements were carried out using the minimum flow rate of 4.99 × 10^−5^ (± 1.70 × 10^−2^) m^3^/s by the pump at a voltage of 4.0 (± 0.1) V. The pre-study with different flow cell arrangements (Figure 5) should prove to control flow velocity and prevent the building of bubbles, as well as the sticking of the particles to the glass surface.

## 4. Data Processing

The spectrometer uses an internal measurement of each laser pulse energy and other system parameters to automatically compensate for any drift or deviation induced by such changes. This ensures high data quality and good traceability.

All measured spectra undergo dark, current, and baseline correction, smoothing (Whittaker–Henderson Second-Order Smoothing) and normalization of the intensity scale. The normalization was based on the laser energy and was performed before averaging repeat measurements. The remaining trends, which could not be corrected by the baseline correction, are corrected by adjusting the offset. This processing step is especially necessary when negative intensities occur. In this study, the band position was determined by fitting Gaussian and Lorentz profiles to enable an assessment of the spectrometer accuracy. The fitting algorithm is based on the “Nonlinear Least Square fitting with Levenberg–Marquardt”.

### Machine Learning

The analysis of spectroscopic data is often challenging. This is due to the complex and diverse nature that recorded spectra exhibit. Chemical substances furthermore show anisotropies in their elastic scattering properties that depend on the orientation of the molecule relative to the laser. While the visual interpretation of recorded spectra is a commonly applied method, their automated classification has been a subject of research for several decades now [23]. Automated classification of spectra yields several advantages. For one, the inherent subjectivity that lies in the visual interpretation is eliminated and instead replaced by a reproducible, mathematical model. Furthermore, slight variations in spectra can be identified much more easily by a suitable algorithm, enabling an automatic classifier to make more nuanced distinctions than a human interpreter could. Finally, the algorithmic classification has the potential to obtain results rapidly and reliably even in continuous processes, making it much more suitable for full-time online monitoring than human interpretation.

Due to the high dimensionality and complex nature of commonly collected Raman spectra, linear models are unsuitable as classifiers in this context. For some time now, the application of artificial neural networks (ANN) to spectral classification problems has proven to be useful on spectra taken from complex mixtures of organic and inorganic compounds [23,43,44]. Especially the ANN variant ‘convolutional neural network’ (CNN) shows some significant advantages over other models, namely improved sensitivity, specificity, and the need for little to no data preparation [23]. CNNs are, therefore, very suitable models to automatically classify spectra taken from various types of samples. In this study, we utilize a novel CNN for data analysis developed by Liu et al. [45]. The authors have shown that the use of CNN can overcome most challenges of classical analysis methods, which need data preparation and signal processing before the actual analysis. Their proposed CNN improves commonly used approaches both in prediction accuracy (in our current CNN, about 97% prediction accuracy on the RRuff test data set) and classification speed. It also eliminates the need for most data preprocessing.

In the initial stage of software development, we reproduced the neural network with the same test data (RRuff minerals data library) and were able to reproduce the results from Liu et al. [45]. In the second stage, our own Raman data for different materials were added. The initial training on the RRuff database serves the purpose of adapting the CNN to Raman spectral data in general since the database provides the large amount of data needed for robust training. The spectra were randomly divided into training (80%), validation (10%) and test (10%) datasets. The training step was done in 500 epochs. The data preprocessing was limited to resampling of the wavelength shifts to values from 1 to 4001, and the intensity values were rescaled to a value range from 0 to 1. If less than 20 spectra were available for any given substance, the data were augmented using the conventional methods of adding noise, adding slight wavelength shifts, and mixing different spectra. The training was done on a NVIDIA RTX2080 Ti with 11 GB of Video RAM. The training with about 30,000 spectra took roughly one hour. The resulting CNN has a total of 2343 output classes consisting of minerals, microplastics, and pharmaceuticals. The classification of unknown spectra with the trained model takes only a few milliseconds on a standard CPU, which makes it suitable for real-time classification directly on the sensor itself.

In our software, we use the machine learning library TensorFlow from Google [46], which enables to load or update the machine learning model by simply deploying a new model file without the need for a software update. This offers additional flexibility to customize and adapt the analysis to new use cases and continuously improve the models.

## 5. Results and Discussion

### 5.1. Results from Nitrates/Nitrites

**Figure 6:**
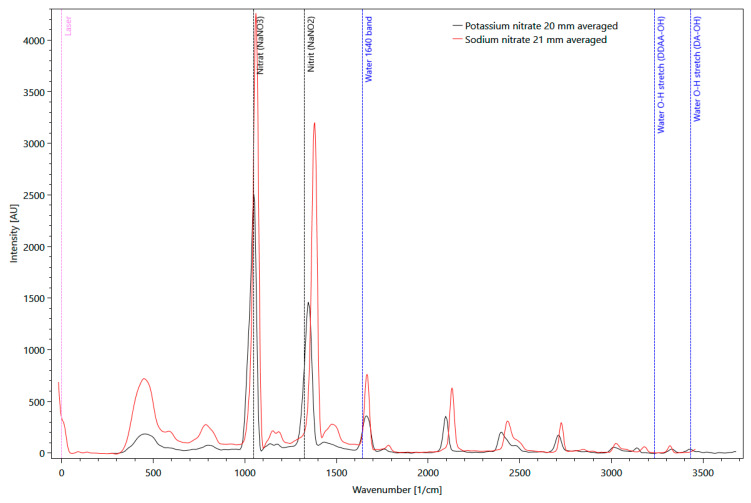
Comparison of pure potassium (black line) and sodium nitrate (red line) Raman spectra, measured as solid matter in a fused silica cuvette, with background information about molecular vibrations of H_2_O (blue dotted lines).

Solid matter Raman measurements with pure potassium (KNO_3_) and sodium nitrate (NaNO_3_) were performed. Several distinct signals are clearly visible in Figure 6 for both substances, with a significantly higher intensity of sodium nitrate compared to potassium. Sodium nitrate shows the maximum signal at a wavenumber of 1044 1/cm. This signal was also used as a reference signal.

**Figure 7:**
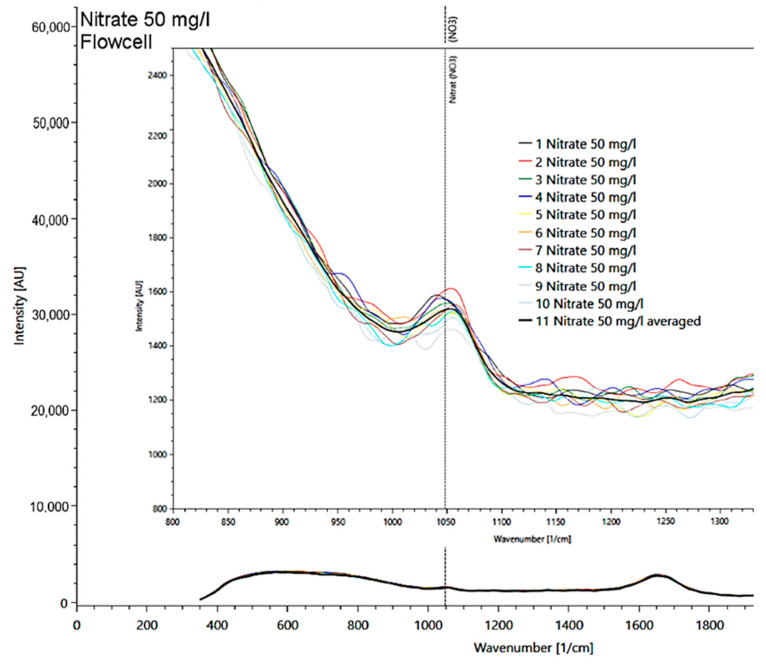
Presentation of the 50 mg/l nitrate spectrum measured in a flow cell with detail enlargement of the 10 measurement repetitions (multicolored lines; average as black line) at the reference signal at 1048 1/cm.

In a solution with a nitrate concentration of 50 mg/l, the main signal at 1048 1/cm, found in measurements of the solid matter, was also clearly visible (Figure 7). Other nitrate peaks could not be identified in the liquid sample due to the strong intensity of the water spectrum, which masks other nitrate peaks.

To overcome this limitation, the capability of the analysis software was extended for subtracting spectral water information obtained from a reference measurement from the measured spectrum.

**Figure 8:**
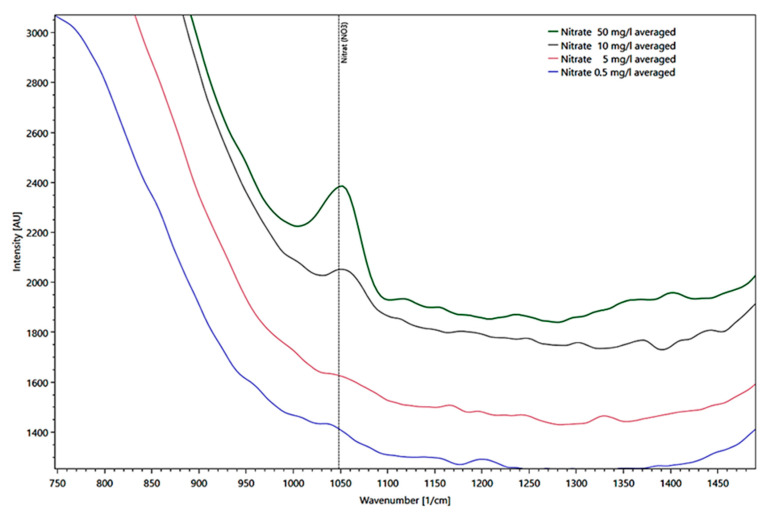
Presentation of different water peaks corrected for nitrate average concentrations from 50 (green line), 10 (black line), 5 (red line), to 0.5 mg/l (blue line) derived from their intensities as Raman spectra, measured in a fused silica cuvette.

The presentation of different nitrate concentrations derived from different intensities measured in a fused silica cuvette at the reference peak at 1048 1/cm is shown in Figure 8 in a detail enlargement of the spectrum.

The same procedure was applied for nitrite with different dilutions in the fused silica cuvette as well as in the flow cell.

In some cases, nitrate signal isolation was difficult due to an unfavorable signal-to-noise ratio or other interfering signals. The concentration was calculated by assuming a Lorentz curve for the reference signal. In addition, the parameters pulse count, laser power, and concentration of the reference measurement were used to obtain the most accurate results possible. After applying the mathematical adjustments to the 50 mg/l nitrite series, the mathematical average value can be adjusted from 57.21 to 51.86 mg/l. In terms of sensitivity, differences in the mg/l range can be recorded. However, the detection accuracy suggests that variations in the decimal range cannot be quantified.

**Figure 9:**
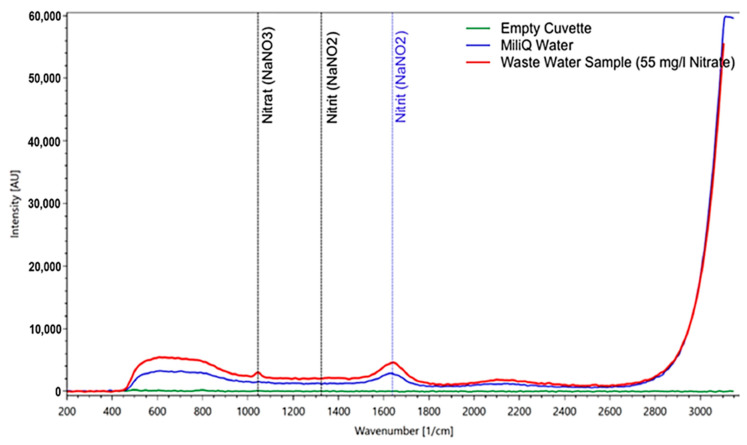
Measurement of a wastewater plant sample (after the biological cleaning stage) containing 55 mg/l of nitrate according to the independent lab analysis. For comparison, the spectrum of pure water and of the empty cuvette are included. The nitrate peak is clearly visible and not obscured by any other substances in the sample.

The first result in a wastewater sample shows that the RPL 200 is able to identify nitrate, even in a solution with a wide variety of dissolved ingredients (Figure 9).

### 5.2. Results of the Pharmaceutical Substances

**Figure 10:**
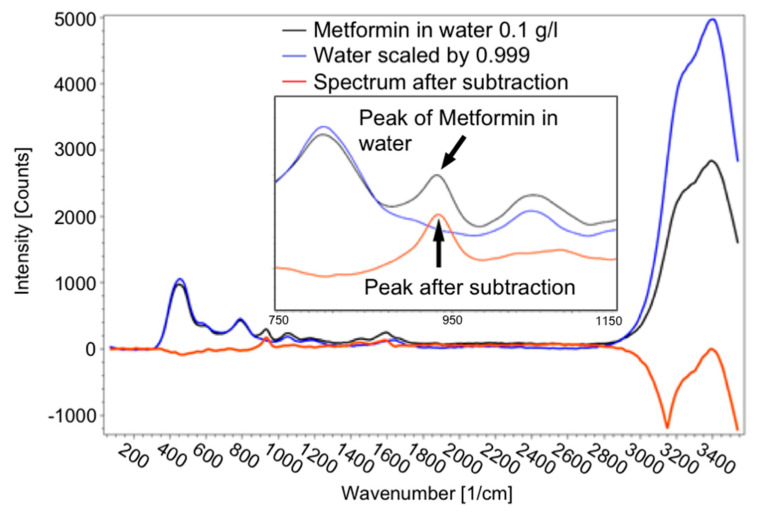
Spectrum of 0.1 g metformin hydrochloride in 1 l water (black line), spectrum of scaled water (blue line) and spectrum after subtraction of the spectrum of water from the solution (red line), as well as a detailed view of the peak of metformin hydrochloride to determine the concentration.

For detecting pharmaceutical substances, which were partly dissolved in ethanol or water, it was inevitably necessary to subtract the ethanol/water spectrum from the spectrum of the dissolved substance according to its proportion in the solution, as illustrated in Figure 10. As long as a peak of the drug is visible in the spectrum, it can be detected. To determine the intensity of the peak, the spectrum of the solvent was subtracted from the spectrum of the solution.

The detection limit for the pharmaceuticals in solution was reached when the signal-to-noise ratio was 3:1. The noise was empirically calculated using the number of slope changes at which the signal increases and subsequently decreases at a wavenumber without a continuous trend. The signal strength was then averaged and used as an approximation for the strength of the background noise. For powdered medication, an overall decrease in signal intensity was measured with increasing numbers of measurements. Thus, the spectral database developed in this study accounts for the measured intensity change over five measurements with 500 pulses.

The Raman spectrum of metformin hydrochloride shows increasing intensities from wavenumbers larger than 400 1/cm. The most intense spectral features were found at 2900–3400 1/cm and, more specifically, 3370 1/cm (largest intensity). The latter peak is likely associated with the asymmetric oscillation of the primary amine [47]. The peaks at 3084 and 3157 1/cm were related to the stretching of N-H [48]. For comparison with the literature data, the spectra measured in this study were corrected by adjusting a Rolling Baseline correction algorithm.

**Figure 11:**
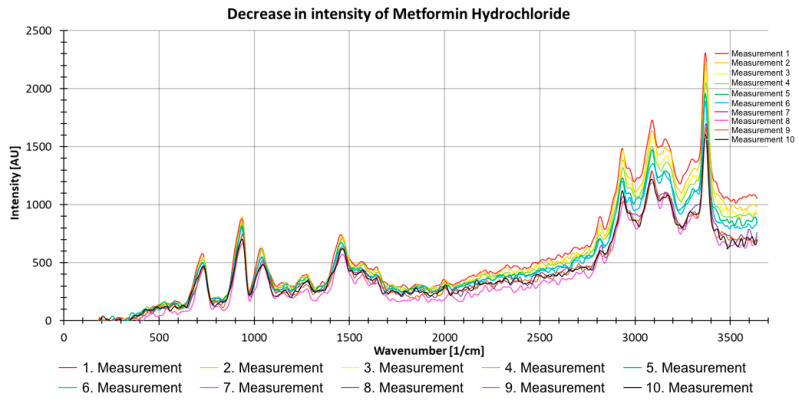
Decrease in intensity of 10 consecutive Raman measurements (with 500 pulses) of metformin hydrochloride as a powder.

Figure 11 shows the results of 10 consecutive measurements with metformin hydrochloride. The decreasing intensity trend with an increasing number of measurements can clearly be seen (Figure 11). The spectra were thus corrected, and all spectra were excited with the same laser energy.

**Figure 12:**
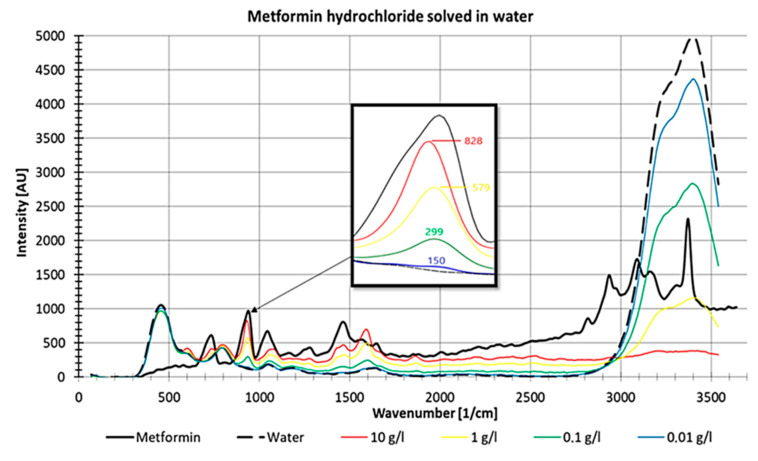
Raman measurements of pure metformin and different concentrations of metformin hydrochloride dissolved in water, measured in a fused silica cuvette with detail enlargement.

For metformin, a non-linear decrease in intensities with decreasing concentrations was observed (Figure 12). In Figure 12, it is also visible that the water peak between 3000 and 3600 1/cm increases with decreasing metformin hydrochloride concentrations, which suggests increasing visibility of the water peak with decreasing substance concentrations.

**Figure 13:**
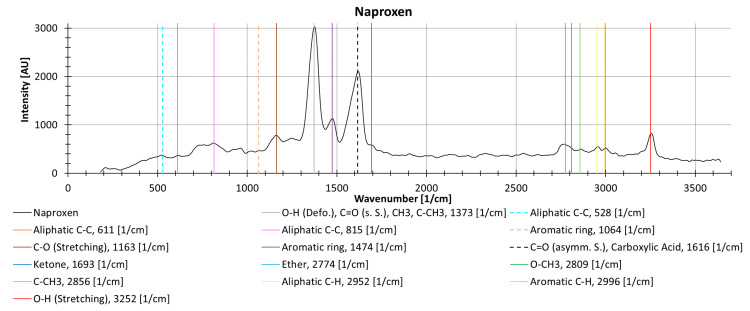
Raman spectrum from naproxen as a solid powder (without cuvette), with information about molecular oscillation and peak identification from the KnowItAll software [47].

The different drugs show different decreasing trends with an increasing number of repeat measurements, possibly due to photodegradation. Naproxen, measured as a solid in powder form without cuvette, shows locally (i.e., individual wavenumbers) more than a 35% decrease between two measurements. The peak at 1377 1/cm (is the deformation vibration of O-H, the symmetrical vibration of C=O, CH_3_, and C-CH_3_) decreases substantially during repeat measurements (37.2%), while the peak at 1619 1/cm (results from the asymmetric oscillation of C=O and the carboxylic acid) decreases by only 9.0% (Figure 13).

**Figure 14:**
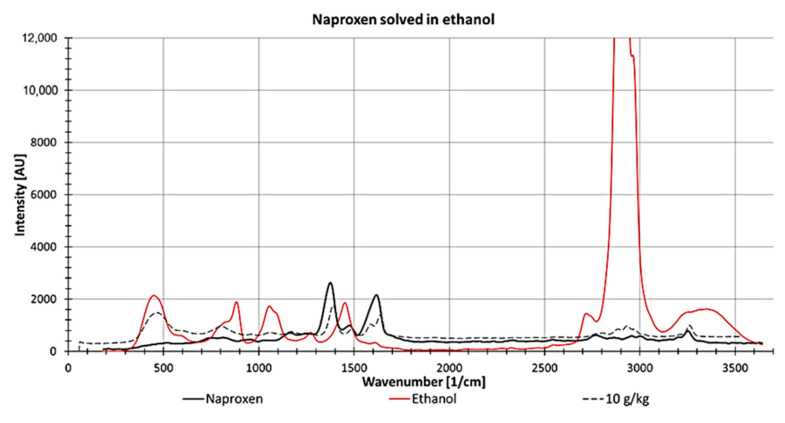
Raman spectrum from naproxen as a solid (black line) and ethanol (measured in a fused silica cuvette, red line) and 10 g naproxen dissolved in 1 kg ethanol (measured in a fused silica cuvette; dotted line).

For determining naproxen concentrations dissolved in ethanol, the peak at 1373 1/cm was used because it showed the lowest spectral perturbation stemming from ethanol and the highest intensity of Naproxen (Figure 14). However, at this wavenumber, both spectra overlap slightly, which increases the complexity of determining the peak height at low concentrations.

**Figure 15:**
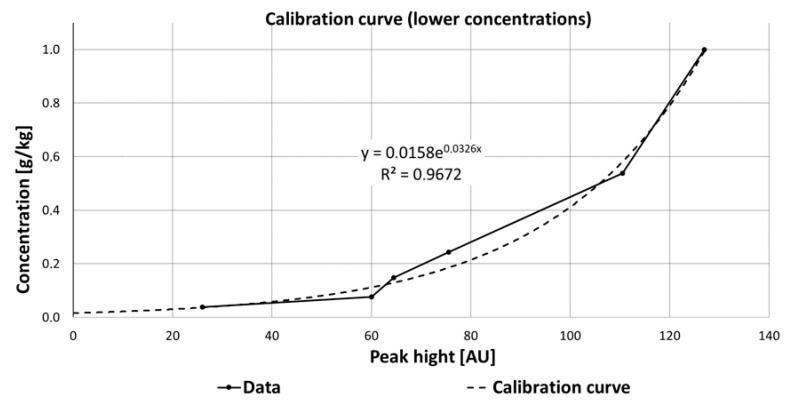
Calibration curve of carbamazepine at lower concentrations, fitted with an exponential function, which is described by the equation y = 0.0158e^0.0326x^.

Regarding the pharmaceuticals and the results of their Raman measurements, it is obvious that a detailed view on the interaction between the substances, their solvents, the measurement set-up (flow cell or cuvette), and possible degradation processes have to be taken into account. For some substances such as carbamazepine, an improved detection was observed at lower concentration (<1 g/kg ethanol). The data obtained for determining the calibration curve at low concentrations of carbamazepine were fitted with an exponential function (y = 0.0158e^0.0326x^). The zero point of the equation and thus the theoretical detection limit is 0.0158 g carbamazepine per 1 kg ethanol. The coefficient of determination R^2^ was 96.7% (Figure 15) and thus significantly larger than for higher concentrations, which varied from 1 to 10 g/kg (R^2^ = 74.6%).

**Figure 16:**
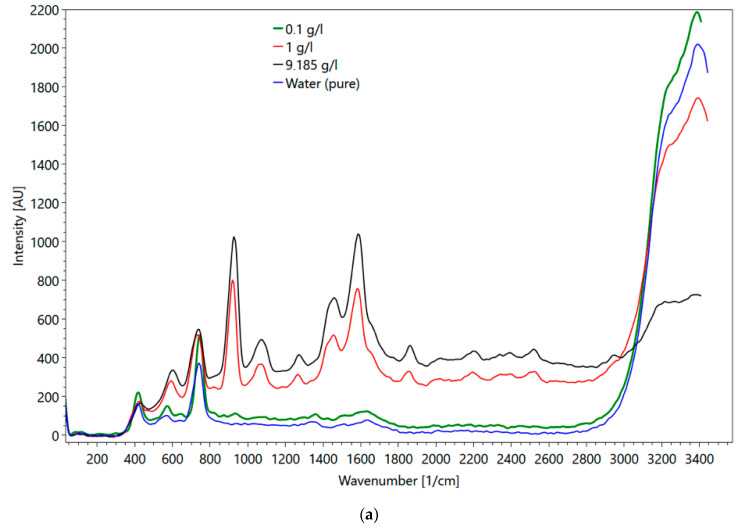

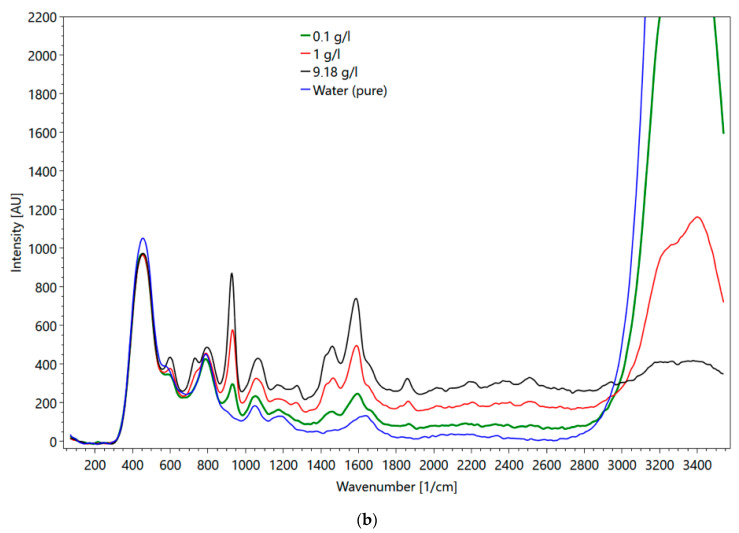
(**a**) Metformin hydrochloride measured in the flow cell with 0.1 (green line), 1 (red line), 9 g/l (black line) and water (blue line); (**b**) metformin hydrochloride measured in the fused silica cuvette at 9 (black line), 1 (red line), 0.1 g/l (green line) and water (blue line).

Metformin hydrochloride, which is soluble in water, was selected for the flow cell experiments. The major objectives of these experiments were (1) to evaluate the impact of the cuvette material (i.e., quartz versus sapphire) on the obtained spectra, and (2) to mimic conditions, which represent transport velocities in a wastewater treatment plant. Figure 16a shows metformin spectra obtained in the flow cell and Figure 16b in a fused silica cuvette. Up to a wavenumber of 800 1/cm, the influence of the different set-ups on the spectra is visible (compare the spectrum of fused silica cuvette in Figure 4d). For wavenumbers larger than 800 1/cm, the peaks of both spectra are the same. One major difference between the spectra of the two set-ups is the intensity of the water peaks (3000–3500 1/cm). Although the water peak in the fused quartz cuvette has approximately twice the intensity of the water peak obtained with the flow cell, the peaks of metformin hydrochloride were less affected, suggesting different sensitivities of the glasses. The maximum metformin hydrochloride intensity measured with the fused quartz cuvette was at a 23 mm sample distance. The intensity of water was at the maximum when the flow cell was directly adjacent to the spectrometer. At higher concentrations, the intensity of the peak for determining the concentration was larger in the flow cell than in the cuvette. At 0.1 g/l the peak height in the flow cell was smaller.

### 5.3. Results of Microplastics (MP)

**Figure 17:**
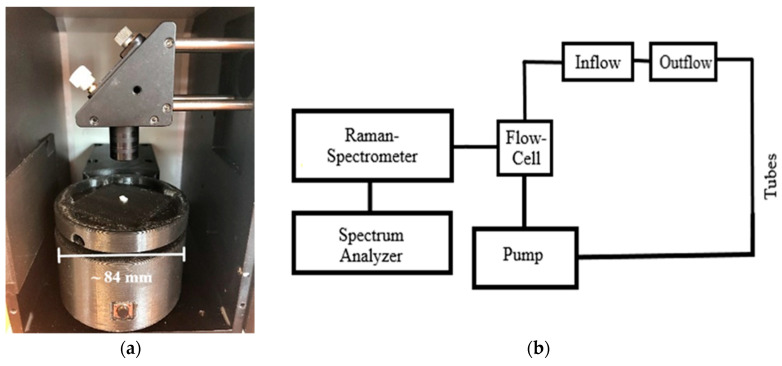
(**a**) Rotary table for the detection of MP particles, (**b**) scheme of the experimental setting for real-time detection of MP in streaming water under different flow behavior.

**Figure 18:**
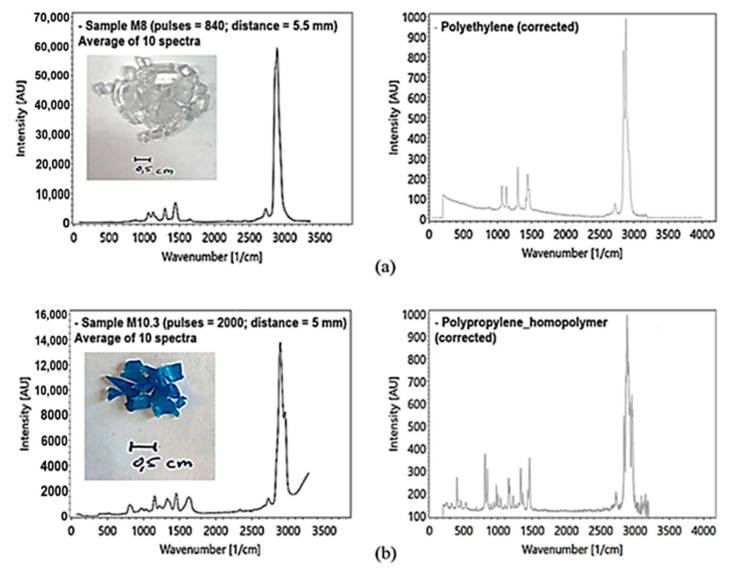
(**a**) PE sample, transparent, Raman measured with the rotary table, averaging of 10 spectra ((**a**) left) with reference spectra of PE ((**a**) right) by [39]; (**b**) PP sample, blue, Raman measured with the rotary table, averaging of 10 spectra ((**b**) left) with reference spectrum ((**b**) right) by [39].

To avoid the influence of inelastic scattering at the silica cuvette glass wall, the polymer recyclates were measured in a modified test arrangement. The analysis of the microplastics was performed using a rotary table (Figure 17a). The height of the rotary table can be adjusted to optimize the focal length in respect of the sample distance to the lens. Polymer recyclates were measured with at least 500 pulses (Figure 18a,b).

The obtained results suggest three factors that affect the data quality: (1) measurement time, (2) sample distance, and (3) the shape and position of the polymer particle. The particle surface led to strong fluctuations in the measurements of the same polymer type and pigmentation.

In addition to a spectral component analysis, the obtained spectra were compared with selected, published reference spectra (Figure 18a,b right) using the KnowItAll spectroscopy database [47]. This comparison showed an 80% agreement with published spectra. This might be related to an incomplete separation of the different microplastic species by the recyclate manufacturer.

In the large flow cell, measurements were obtained while the flow behavior was varied by adjusting the flow velocity. Under stationary conditions, particle detection required the particle to be in focus. Thus, limitations in detection are mainly associated with too fast flow rates (with the formation of bubbles) and a too-small laser cross-section. To improve the detection capabilities, the flow rate was, controlled and MP particles were used that showed Raman bands, which are unaffected by the water band. In addition, the concentration of particles was increased.

**Figure 19:**
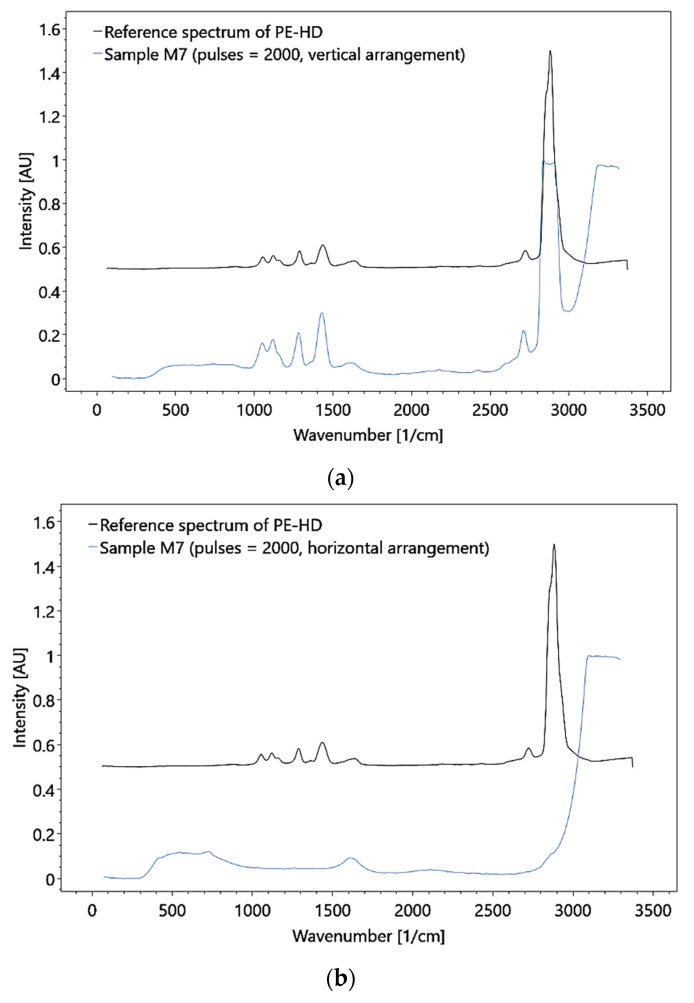
Raman analysis of the HDPE sample in the large flow cell, under (**a**) vertical and (**b**) horizontal arrangement, with a particle concentration of 1414.7 particles per 270 ml and 0.5 mm for the particle size.

These adjustments allowed the detection of polymer HDPE MP with a particle size of 0.5 mm at a flow rate of 3.0 × 10^−1^ (± 1 × 10^−2^) m/s and a mass concentration of 1.11 × 10^−3^ g/ml (i.e., a particle concentration of 1414.7 particles per 270 ml, Figure 19). Although the test results demonstrate the feasibility of MP detection in principle, the utilized concentrations do not represent natural conditions. Moreover, only particles hit by the laser could be detected, and thus, the measurements were subjected to a statistical probability.

## 6. Conclusions and Outlook

We tested the feasibility of using deep UV Raman spectroscopy in combination with artificial intelligence software for environmental monitoring. In this initial study, we selected substances based on their environmental impact (i.e., nitrate, pharmaceuticals, microplastics).

Initial results gained from a series of experiments with a variety of substances and a bundle of different experimental set-ups measured suggest that the reliability for detecting and monitoring the selected substances in water with Raman spectroscopy suffers from a series of limitations. A major limitation is the low sensitivity (i.e., the detection of lower concentrations in the µg or ng scale). MP measurements are limited by the probability of particles in the focal point of the laser at low concentrations or at high flow velocity.

Although the CNN performs better for data classification than more classical approaches such as Pearson correlation or other mathematical algorithms of pure substances with high signal-to-noise ratios, our first measurements of dissolved pharmaceuticals show that the main challenge will be to get a useable signal at all for the very low concentrations of pharmaceuticals in actual wastewater or groundwater samples. Therefore, more measurements are needed to assess the performance and limits of the CNN when provided with very low signal-to-noise ratio data. However, current developments focus on the fusion of Raman and fluorescence measurement to combine the high sensitivity of fluorescence measurement with the high specificity of Raman.

First results on wastewater samples show that the RPL 200 is able to detect nitrate ions even in the presence of a wide variety of other ingredients.

The additional data gained from the sensor fusion approach may help for better training of the machine learning and to make predictions and classification results more robust by allowing cross-validation with independent sensor data in real-time.

## Data Availability

Data sharing not applicable.
